# Mutagenesis-Mediated Decrease of Pathogenicity as a Feature of the Mutant Spectrum of a Viral Population

**DOI:** 10.1371/journal.pone.0039941

**Published:** 2012-06-28

**Authors:** Marta Sanz-Ramos, Teresa Rodríguez-Calvo, Noemí Sevilla

**Affiliations:** 1 Centro de Investigación en Sanidad Animal, Instituto Nacional de Investigación y Tecnología Agraria y Alimentaria (CISA-INIA), Valdeolmos, Madrid, Spain; 2 Centro de Biología Molecular Severo Ochoa, Consejo Superior de Investigaciones Científicas, Campus de Cantoblanco, Madrid, Spain; German Primate Center, Germany

## Abstract

**Background:**

RNA virus populations are heterogeneous ensembles of closely related genomes termed quasispecies. This highly complex distribution of variants confers important properties to RNA viruses and influences their pathogenic behavior. It has been hypothesized that increased mutagenesis of viral populations, by treatment with mutagenic agents, can induce alterations in the pathogenic potential of a virus population. In this work we investigate whether mutagenized foot-and-mouth disease virus (FMDV) populations display changes in their virulence in mice.

**Methodology and Principal Findings:**

FMDV C-S8c1 was passaged in BHK cells in the presence of the mutagenic agent ribavirin. Decline in viral titer and viral RNA progeny was observed in the first passage, fluctuating around a constant value thereafter. Hence, the specific infectivity remained stable during the passages. The viral population harvested from passage 9 (P9 R) showed decreased virulence in mice, with a lethal dose 50 (LD_50_) >10^4^ PFU, as compared with LD_50_ of 50 PFU of the parental population FMDV C-S8c1. This decrease in virulence was associated to a 20-fold increase in the mutation frequency of the P9 R population with respect to C-S8c1. Interestingly, individual biological clones isolated from the attenuated population P9 R were as virulent as the parental virus C-S8c1. Furthermore, a mixed population of C-S8c1 and P9 R was inoculated into mice and showed decreased virulence as compared to C-S8c1, suggesting that population P9 R is able to suppress the virulent phenotype of C-S8c1.

**Conclusion:**

Ribavirin-mediated mutagenesis of an FMDV population resulted in attenuation *in vivo*, albeit a large proportion of its biological clones displayed a highly virulent phenotype. These results, together with the suppression of C-S8c1 by mutagenized P9 R population, document a suppressive effect of mutagenized viral quasispecies *in vivo*, and suggest novel approaches to the treatment and prevention of viral diseases.

## Introduction

RNA viruses replicate as complex mutant distributions termed viral quasispecies [reviews in [1]–[4]. This means that replicative ensembles of non-identical genomes are generated within infected cells, and that different variants can coinfect cells as replication proceeds. Several studies have documented that internal interactions among viral gene products of the same replicative ensemble (or mutant spectrum) may result in either survival of defective genomes by complementation or in suppression of higher fitness virus by lower fitness mutant distributions [2], [4]–[8]. Complementation among components of a viral quasispecies replicating under standard mutation rate may also underlie the observation that replicative fitness (relative capacity to produce infectious progeny) of individual clones is, on average, lower than the fitness of the entire (uncloned) populations from which the clones were isolated [9], [10].

Several important biological properties of RNA viruses are heavily influenced by the complexity (number of different genomic sequences and average number of mutations per genome) and composition of mutant spectra. Among them, the presence of memory in viral quasispecies, the rate of disease progression, the response to antiviral treatments, and the pathogenic potential of a virus, as documented with the decreased neuropathology displayed by a high fidelity mutant of poliovirus that produces a less complex mutant spectrum than the wild-type virus [11], [12]. High fidelity mutant viruses constitute a new generation of candidate attenuated vaccines ([13]–[14]; reviewed in [2], [4]). In cell culture, the interaction between two foot-and-mouth disease virus (FMDV) subpopulations, which had evolved from a single parental genome, modulated the cell killing capacity of the population [15]. In a study of infection of adult mice with FMDV, the virus isolated from pancreas was highly attenuated for mice although the virus showed indistinguishable consensus genomic nucleotide sequence and mutant spectrum complexity compared to virulent virus isolated from sera of the same animals [16]. Thus, mutant spectra of viral populations may encode virulence determinants not readily associated with the complexity of the mutant spectrum or with a specific, dominant genome sequence. Overall, these observations reinforce the concept that it is an ensemble of genomes –rather than individual genomes within the ensemble– the one that determines virus behavior. Interestingly, this corresponds to the tenet of quasispecies theory of the mutant spectrum being the target of selection [17]–[19]. In quasispecies theory, selection of ensembles was due to the high connectivity of genomes in sequence space, and in the case of viruses the selection is prompted not only by connectivity in sequence space but also by the interactions among gene products of related viral genomes at a functional level [4].

Interfering interactions within mutant spectra may also be one of the mechanisms of virus extinction by elevated mutation rates in a process that has been termed lethal mutagenesis or transition into error catastrophe [20]–[25]. Defective genomes, generated by mutation of the standard viral genomes when viral replication occurs in the presence of a mutagenic agent, may interfere with replication of infectious virus, thereby contributing to viral extinction [26]–[29]. Unless mutagen-resistance mutations are selected, passage of a virus in the presence of a mutagenic agent often results in virus extinction accompanied of logarithmic decreases of specific infectivity (measured as PFU/viral RNA molecules), and with an invariant consensus genomic nucleotide sequence [26]–[30]. Other than examining the events associated with extinction at the level of quasispecies complexity and its variations [31], no other biological effects of enhanced mutagenesis on the behavior of RNA viruses have been studied.

Mutagenized viral populations, by virtue of containing a broader mutant repertoire, might manifest an enhanced adaptive and pathogenic potential as observed with poliovirus quasispecies replicating under standard (or lower than standard) mutation rates [11], [12]. Alternatively, mutagenized quasispecies may display reduced adaptability due to the presence of defectors [28]–[30], [32], [33]. To investigate whether mutagenized FMDV populations showed an increase or a decrease of virulence we used the FMDV C-S8c1-adult C57BL/6 mice system, which displays features of FMDV infection of natural hosts (cloven-hoofed animals) [16], [34]. FMDV is a positive ssRNA virus, which belongs to the *Picornaviridae* family. It encodes for low fidelity polymerases, known as 3D, that has been shown to incorporate mutagenic nucleotides, such as ribavirin triphosphate [35]. FMDV C-S8c1 was passaged in BHK-21 cells in the presence of ribavirin to increase the complexity of the mutant spectrum of the viral population and, equivalent amounts of infectious virus from different passages were tested for their virulence in mice. The results show that while the specific infectivity did not decrease significantly in the course of passages in the presence of ribavirin, lethality for mice decreased with passage number. Interestingly, five biological FMDV clones isolated from the passaged population with decreased virulence, showed high lethality for mice. This implies that the attenuated phenotype was conferred by the viral population as a whole, and that the mutant spectrum included biological clones, which individually depicted high virulence.

## Results

### Passage of FMDV C-S8c1 in the presence of ribavirin results in sustained specific infectivity

**Figure 1:**
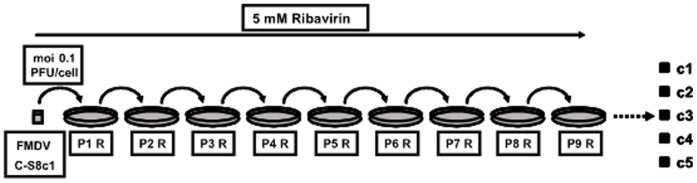
Scheme of passages of FMDV C-S8c1 in BHK-21 cells in the presence of ribavirin. FMDV C-S8c1 was subjected to nine passages in BHK-21 cells in the presence of 5 mM of ribavirin. The viral population isolated from each passage is identified as a P followed by the passage number and R. The initial viral infection was performed at a moi of 0.1 PFU/cell, and subsequent infections were carried out with 1/10 of the volume of the previous passage (moi in the range of 0.01–0.1 PFU/cell). Five biological clones, represented as black squares, were isolated from viral population P9 R by dilution and plating on BHK-21 cell monolayers, as indicated with a dotted line on the right.

**Figure 2:**
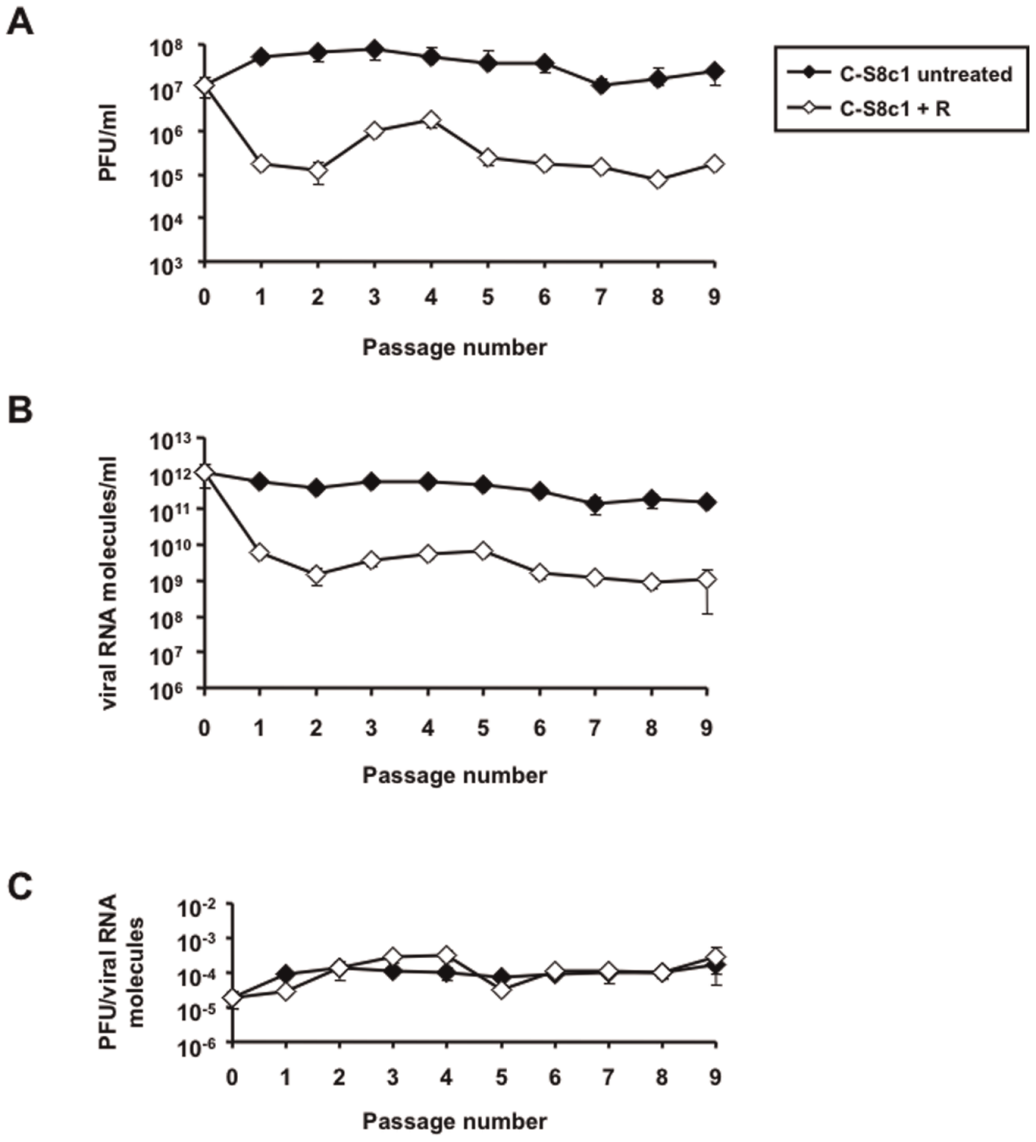
Viral titers, RNA molecules and specific infectivity of C-S8c1 along passages in BHK-21 cells. Viral titer (A), number of viral RNA molecules/ml (B), and specific infectivity (C) values of C-S8c1 passaged in the presence of 5 mM ribavirin are compared to those obtained in the absence of the drug. Viral titer is expressed as PFU/ml. The specific infectivity represents the ratio between viral titer and number of RNA molecules, shown in A and B. Procedures for the infections with FMDV, and determinations of infectivity and viral RNA are detailed in Materials and Methods.

Biological clone FMDV C-S8c1 [36] was passaged in BHK-21 cells in the presence of 5 mM ribavirin (Figure 1). Infectivity and viral RNA levels were determined at each passage (Figure 2A and B). After a decline in titer and viral RNA progeny in the first passage in the presence of ribavirin, viral titers and RNA levels remained approximately constant until at least passage 9. As a consequence, no clear trend towards either an increase or decrease in specific infectivity was manifested, despite some variations during the nine serial passages (Figure 2C). Unaffected high titers and RNA levels were observed in untreated FMDV C-S8c1 along passages resulting likewise in constant specific infectivity values (Figure 2).

### FMDV virulence for mice decreases with the number of passages in the presence of ribavirin

**Figure 3:**
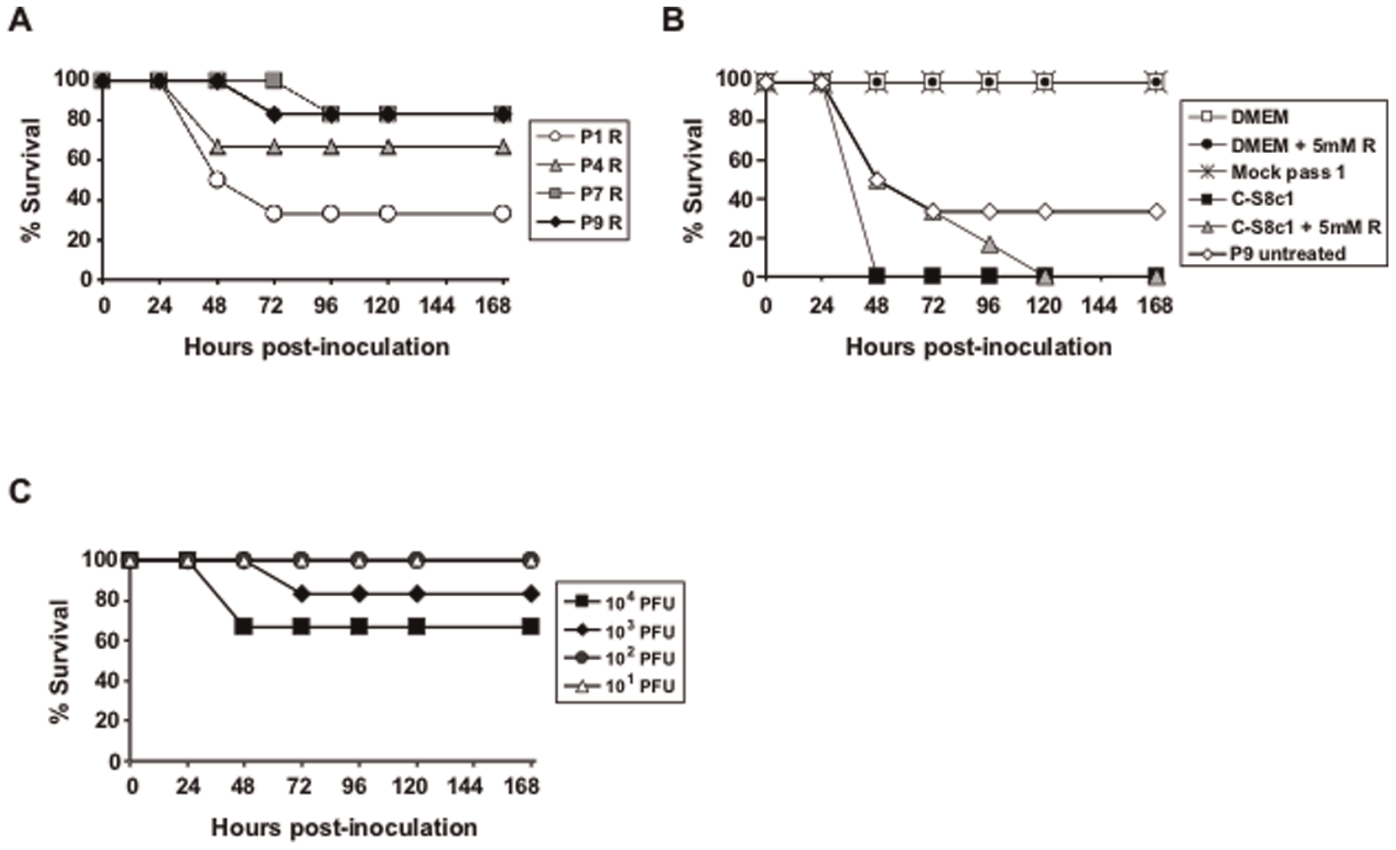
Virulence in mice of FMDV C-S8c1 passaged in BHK-21 cells in the presence of ribavirin. In A) and B) groups of six mice were inoculated into the footpad with 10^3^ PFU of each viral population or culture media, as indicated in the boxes. Percentage of survival of the inoculated mice during a follow-up period of 168 hours is represented. Samples are shown in two separate graphics for a better visualization. A) Percentage of mice that survived following inoculation with viral populations recovered from passages 1, 4, 7 and 9 of FMDV C-S8c1 in BHK-21 cells in the presence of 5 mM ribavirin (virus nomenclature is according to Figure 1). B) Percentage of mice that survived following inoculation with several control samples. Positive controls correspond to mice infected with FMDV C-S8c1, a mixture of FMDV C-S8c1 and 5 mM of ribavirin, or the viral population resulting from nine passages in BHK-21 cells in the absence of ribavirin (P9 untreated). Negative controls include mice inoculated with DMEM (the medium in which viruses were diluted), DMEM with 5 mM ribavirin, or the supernatant of a non-infected BHK-21 cell culture maintained during 24 hours in DMEM 5 mM ribavirin (Mock passage 1). C) LD_50_ of viral population P9 R. Groups of six mice were inoculated into the footpad with the number of PFU of population P9 R indicated in the box. The percentage of mice that survived was calculated over a follow-up period of 168 hours. Procedures are detailed in Materials and Methods.

FMDV C-S8c1 is lethal for C57BL/6 mice, with a lethal dose 50 (LD_50_) value of 50 PFU [34]. To investigate whether passage of FMDV C-S8c1 in the presence of ribavirin decreased the lethality of the virus for mice, equal PFU (10^3^) of FMDV C-S8c1 or P1 R, P4 R, P7 R and P9 R were inoculated into mice and the percentage of animals that survived was determined (Figure 3). Controls included animals inoculated with culture medium (DMEM) with or without ribavirin, C-S8c1 in the presence of ribavirin, and C-S8c1 passaged 9 times in the absence of ribavirin (P9 untreated). Virulence of P9 R and P7 R, calculated as the percentage of survival of inoculated mice, was significantly lower than that of either the parental virus FMDV C-S8c1, or the mixture of FMDV C-S8c1 and 5 mM ribavirin (Logrank Test; p = 0.001). Thus, despite the virus maintaining a relatively constant specific infectivity (Figure 2C), its pathogenicity decreased with the number of passages in the presence of ribavirin. The different controls used indicated that passage in the presence of ribavirin is a necessity for the decrease of mouse lethality. The survival of mice inoculated with 10^1^ to 10^4^ PFU of FMDV P9 R indicated that the LD_50_ for this virus population was higher than 10^4^ PFU, a value which represents at least 200-fold lower lethality than that of C-S8c1 (Figure 3C) [34].

### Virulence is a collective property of population FMDV P9 R

**Figure 4:**
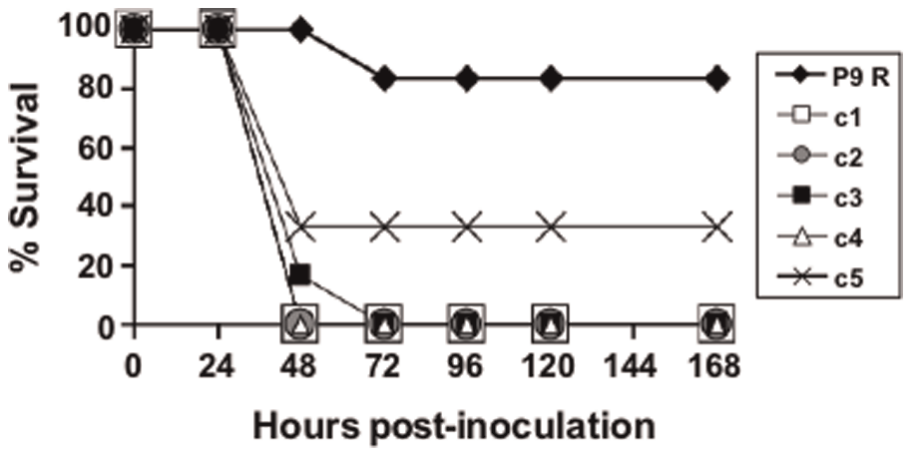
Lethality of biological clones isolated from population P9 R. Groups of six mice were inoculated into the footpad with 10^3^ PFU of P9 R or of each of the 5 biological clones (c1 to c5) obtained as plaque-isolates from population P9 R. The nomenclature of the virus corresponds to that described in Figure 1. Percentage of survival of the inoculated mice during a follow-up period of 168 hours is indicated. Procedures are detailed in Materials and Methods.

To investigate whether the low virulence of population P9 R reflected the virulence of the majority of viable (plaque-forming positive) clones that compose the population, virus from five biological clones derived from population P9 R was obtained by dilution and plating on BHK-21 cell monolayers. Each of the clones analyzed showed higher virulence in mice than the parental viral population P9 R from which they were isolated (Figure 4). In particular, biological clones c1, c2, c3, and c4 displayed significantly higher virulence than P9 R (Logrank Test, p = 0.001). Thus, population P9 R included highly virulent virus whose capacity to kill mice was not manifested in the context of the entire viral population. The reduced virulence of P9 R is a collective property of the mutagenized quasispecies.

### A single mutation distinguishes FMDV population P9 R from C-S8c1

**Figure 5:**
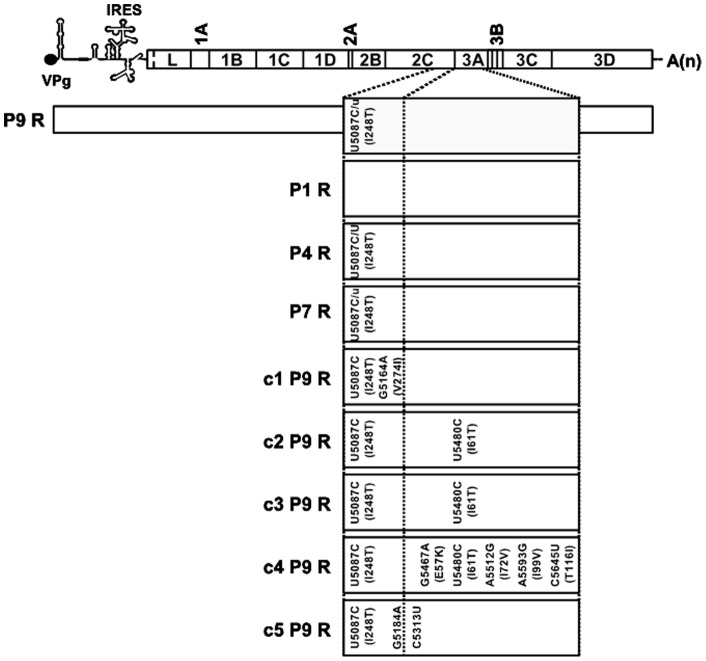
Complete genomic consensus nucleotide sequence of population P9 R and partial sequence of biological clones. A schematic representation of FMDV genome is displayed at the top of the figure [based on [64]–[66]. The entire genomic sequence of FMDV population P9 R and partial sequences, comprising residues 4945 to 5677 of FMDV populations P7 R, P4 R and P1 R, and P9 R-derived clones c1, c2, c3, c4 and c5 were determined. Virus nomenclature is that of Figure 1. Nucleotide substitutions relative to the parental virus C-S8c1 are represented inside the boxes below the corresponding genomic region in which they are found. For non-synonymous mutations the corresponding amino acid replacement is given in parenthesis. An equivalent mixture of C (parental) and U (mutant) nucleotides in the genomic position 5087 is represented by C/U; a higher proportion of C than U (about 75% C) is indicated as C/u. Substitution C5087U leads to amino acid replacement I248T in viral protein 2C. Residue numbering is accordingly to [61].

To identify whether some dominant mutations distinguished population FMDV P9 R from its parental biological clone C-S8c1, the full genome sequence of P9 R was determined, and compared with that of C-S8c1 [37]. In the consensus sequence of P9 R, the only mutation that was present as a mixture with the wild-type nucleotide was U5087C, which gives rise to amino acid substitution I248T in viral protein 2C. This mutation was not detected after the first passage in the presence of ribavirin (P1 R) and its proportion varied in later passages in the presence of ribavirin. Even though it did not reach complete dominance in population P9 R, it was present in each of the five biological clones that displayed high virulence for mice (compare Figures 4 and 5). In addition, we have previously shown that C-S8c1 infectious clone harboring the mutation I248T in 2C displays similar virulence to that of the wild-type sequence clone in infections in mice [16].

The ribavirin-resistance mutations previously described in FMDV and poliovirus map in the viral polymerase (3D) (P44S, G62S, P169S and M296I; residues are numbered according the 3D of FMDV) [38]–[42]. Although the consensus sequence of P9 R did not show any mutation in the 3D-coding gene, we analyzed this region (nucleotides 6800 to 7773; amino acids 65 to 453) in 30 molecular clones from population P9 R. The results (Table 1) revealed 37 different mutations, 10 of which were repeated in two or more clones. There were a total of 14 amino acid substitutions, although none corresponding to the previously described ribavirin-resistance mutations in FMDV [38], [39], [41].

**Table 1:** Mutations identified in the 3D coding region in the mutant spectrum of the population passaged in the presence of ribavirin P9 R and the parental FMDV population C-S8c1.

Viral population^a^	Nucleotide substitution^b^	Amino acid replacement^c^	Molecular clone number^d^
**C-S8c1**	C7407U	---	C26
	U7658G	---	C14
**P9 R**	U6846C	---	C21
	C6881U	T91M	C17
	G6882A	---	C5
	G6909A	---	C16
	G6925A	D106N	C8
	G6949A	D114N	C20
	C6978U	---	C9, C12, C18, C22, C25
	G7027A	G140R	C4
	C7046U	A146V	C6
	A7061C	E151A	C26
	U7083C	---	C11, C23
	C7086U	---	C16, C4
	G7120A	E171K	C2
	U7131C	---	C3
	C7155U	---	C8
	U7176C	---	C7, C10, C16
	A7212G	---	C4
	C7237U	Q210Stop	C15
	G7324A	V239M	C14
	G7326A	---	C13
	C7350U	---	C1
	C7353U	---	C27
	G7452A	---	C21
	C7458U	---	C12, C22, C25
	G7465A	E286K	C27
	G7486A	E293K	C11, C23
	G7489A	G294S	C21, C4
	G7584A	---	C4
	G7585A	V326I	C1
	C7623U	---	C27
	C7641U		C12, C22, C25
	U7650C	---	C4
	C7651U	---	C7, C10, C16
	C7665U	---	C12, C22, C25
	C7697U	T363I	C3
	A7699G	I364V	C2
	C7755U	----	C4

a Population P9 R is that derived from passaging FMDV C-S8c1 nine times in the presence of 5 mM ribavirin, as detailed in the legend for Figure 1 and in Materials and Methods. C-S8c1 is the parental population used to initiate the passages.

b The mutations observed in the mutant spectrum of P9 R and C-S8c1 are indicated. The total number of nucleotides sequenced, mutation frequency and Shannon entropy are given in Table 2.

c Amino acid substitutions deduced from the mutations listed in the second column. A dash line means a synonymous mutation.

d The molecular clones have been numbered to identify linked and repeated mutations.

One of the mutations led to a termination codon within the 3D-coding region (C7237U, CAG to UAG), suggesting that replication in the presence of ribavirin might have increased the proportion of defective genomes in population P9 R (see Discussion). The number of mutations scored in population P9 R represented an increase of 20-fold in the minimum mutation frequency, 31-fold in the maximum mutation frequency, and 10-fold in Shannon entropy, relative to the parental C-S8c1 population (Table 2). Passaging of C-S8c1 in BHK-21 cells in the absence of ribavirin has been previously shown to have little effect on the mutation frequency of the viral population [33], [43]. It has been described that mutation frequencies in the 3D coding region of C-S8c1 passaged four or 25 times in BHK-21 cells in the absence of ribavirin are 2.2×10^−4^ and 2.8×10^−4^ respectively, 6.4 and 5.0 times lower than the mutation frequency of P9 R (1.4×10^−3^, Table 2). The distribution of mutation types in the mutant spectrum of P9 R revealed a great dominance of C→U and G→A mutations (78.3% of the total) (Table 3), as expected from the mutagenic action of ribavirin on FMDV [38], [41], [44]. Thus, replication of FMDV C-S8c1 in the presence of high ribavirin concentrations produced an extremely complex viral population that was highly attenuated for mice, despite including virulent biological clones.

**Table 2:** Mutant spectrum complexity of P9 R and C-S8c1.

Viral population^a^	Genomic region^b^	Number of clones analyzed	Number of nucleotides^c^	Mutation frequency		*S_n_* f
				minimum^d^	maximum^e^	
**C-S8c1**	3D (6800–7773)	30	29220	6.8×10^−5^	6.8×10^−5^	0,09
**P9 R**	3D (6800–7773)	27	26298	1.4×10^−3^	2.1×10^−3^	0,9

a The viral populations are those described in Figure 1.

b The numbering of FMDV genomic residues is according to reference (Escarmís et al. 1999).

c Number of nucleotides sequenced in each viral population.

d The minimum mutation frequency is the number of different mutations divided by the total number of nucleotides sequenced for each viral population.

e The maximum mutation frequency is the total number of mutations divided by the total number of nucleotides sequenced for each viral population

f *S_n_*, indicates normalised Shannon entropy, that is a measure of the proportion of different sequences in a distribution. It was calculated as described in Materials and Methods.

**Table 3:** Types of mutations found in the mutant spectra of P9 R and C-S8c1.

Viral population^a^	Transitions^b^					Transversions^b^		
	U→C	C→U	G→A	A→G	Total	U→G	A→C	Total
**C-S8c1**	0	1 (50%)	0	0	1	1(50%)	0	1
**P9 R**	5 (13,5%)	15 (40,5%)	14 (37,8%)	2 (5,4%)	36	0	1 (2,7%)	1

a The viral populations are according to Figure 1.

b The mutations are those found in the analysis of the mutant spectra of C-S8c1 and P9 R described in Table 1. The percentage of each type of mutation in relation to the total number of mutations found in the same population is shown in brackets. Transversions C→G, G→C, U→A, A→U, G→U and C→A were not observed in the analysis.

### FMDV C-S8c1 is suppressed by the mutagenized quasispecies P9 R

**Figure 6:**
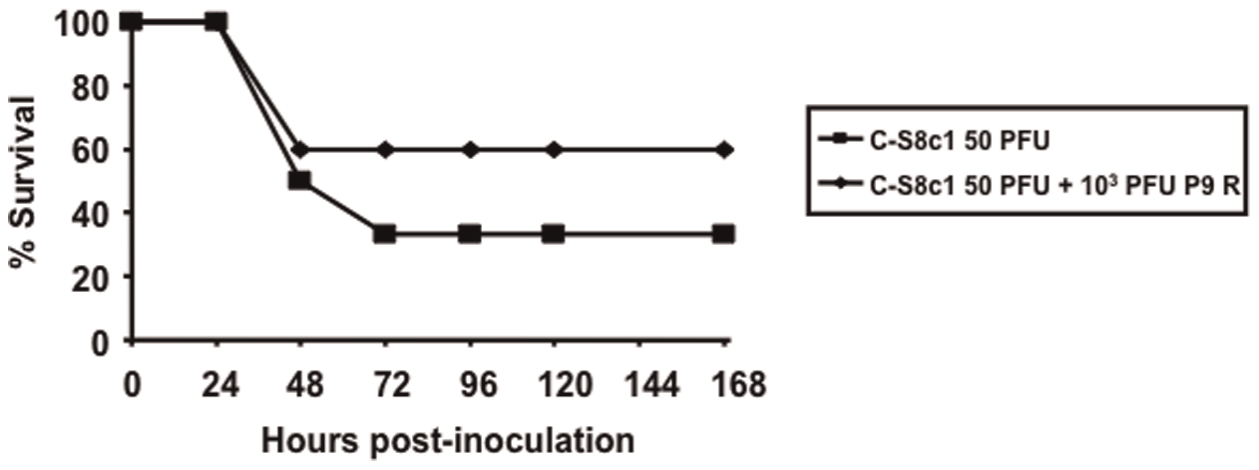
Suppressive effect of the mutagenized population P9 R on FMDV C-S8c1. Two groups of mice were inoculated subcutaneously with either 50 PFU of C-S8c1 (six mice) or a mixed population comprising 50 PFU of C-S8c1 and 10^3^ PFU of P9 R (10 mice). The nomenclature of the virus is as described in Figure 1. Mice were monitored during a follow-up period of 168 hours and the percentage of survival in each group is represented.

In order to determine if the viral population P9 R, highly attenuated in mice, can suppress the parental virus C-S8c1 *in vivo*, groups of adult mice were inoculated with either a mixed population of C-S8c1 and P9 R at a ratio 1∶20 (50 PFU of C-S8c1+10^3^ PFUs of P9 R), or C-S8c1 alone (50 PFU). The percentage of survival during a follow-up period of 168 hours was quantified (Figure 6). C-S8c1 alone displayed higher virulence than C-S8c1 mixed with the complex population P9 R, although it did not reach significance. This result suggests that the complex P9 R population is able to partially suppress the highly virulent C-S8c1 in infections *in vivo*.

## Discussion

Quasispecies dynamics has as one of its major biological implications that viral pathogenesis may be determined by ensembles of genomes that populate the mutant spectrum rather than by a specific mutant type [11]–[13], [45]. In the present study we have examined the behavior of a ribavirin-mutagenized FMDV population with regard to lethality for adult mice, an animal model previously validated as surrogate for the prohibitive large animal infections with FMDV [34]. Previous studies on FMDV mutagenesis in cell culture indicated that heavily mutagenized viral populations interfered with the replication of the standard FMDV genomes [26], [33]. Interference was further documented with specific FMDV mutants that included defined amino acid substitutions either in the capsid or in the polymerase, and that had as a common trait being competent in RNA replication [29]. Ribavirin has been shown to be mutagenic for a wide range of RNA viruses including FMDV, which typically results in a decrease of virus infectivity and occasionally the extinction of the virus population [7], [44], [46], [47] Interference by mutants generated by ribavirin played a role in viral extinction and was one of several determinants rendering sequential administration of an inhibitor followed by a mutagen advantageous relative to the corresponding combination treatment [28]. The results with FMDV are in agreement with those obtained with the arenavirus lymphocytic choriomeningitis virus (LCMV) with a similar experimental design, and support the participation of defective genomes in virus extinction [27], [47], [48].

The present study addresses whether an FMDV population that was mutagenized by passaging the virus in the presence of ribavirin in BHK-21 cells could manifest some alteration of virulence *in vivo*, as determined with a mouse model for FMDV [16], [34]. Population P9 R displayed a significant increase in mutant spectrum complexity (Table 2), but the specific infectivity was not significantly altered relative to the initial biological clone (Figure 2), suggesting that the population did not initiate a transition towards extinction. The reason for the sustained infectivity of biological clone C-S8c1 in the presence of 5 mM ribavirin is unknown, but substitution I248T in 2C might play a role. It has been previously described that this substitution in the context of a ribavirin-resistant FMDV C-S8c1 triple mutant expressing a polymerase with substitutions P44S, P169S and M296I increased the fitness of the triple mutant in the presence of 5 mM ribavirin [38]. Therefore, I248T in 2C together with constellations of mutations in 3D or other viral proteins may limit the sensitivity of the population to ribavirin. The mutations that might be responsible for a decreased sensitivity to ribavirin remain to be determined but they are not those mutations in 3D previously identified as conferring FMDV resistance to ribavirin [38], [39], [41]. In particular, the mutant spectrum of P9 R included a ratio of (C→U)+(G→A)/(U→C)+(A→G) transitions of 4.14, a value which corresponds to the ratio previously observed upon passage of pMT28 in the presence of ribavirin [38]. A ratio of 4.14 excludes the presence of dominant amino acid substitutions that could modulate the types of transition types induced by ribavirin therefore favoring viral survival, as previously documented with amino acid P44S in 3D of FMDV [49]. The different behavior of FMDV C-S8c1 and the progeny of molecular clone pMT28 regarding replication under high ribavirin concentrations is presently under investigation.

Despite a stasis in specific infectivity values (based on infectivity determined with a plaque assay using BHK-21 cells), the progressively complex FMDV population showed a gradual decrease of virulence in mice, manifested already after a single virus passage in the presence of ribavirin (Figure 3). The decrease in virulence cannot be attributed to the presence of some ribavirin contaminating the viral population because the inclusion of 5 mM ribavirin in a C-S8c1 preparation resulted in no survival of mice at 120 h to 168 h p.i. P9 R showed very limited virulence, with more than 80% survival at 72 h to 168 h p.i. (Figure 3). Interestingly, however, most of the biological clones isolated from P9 R displayed as high virulence in mice as the lethal clone C-S8c1 (Figure 4). This suggests that the virulence of a large proportion of plaque-forming viruses was suppressed by the mutagenized populations that surrounded them. Furthermore, P9 R was shown to partially suppress the parental virus C-S8c1 (Figure 6). Suppressive effects of mutant spectra on high fitness variants have been previously documented both in cell culture and *in vivo* [6], [50]–[53]. In the case of ribavirin-mutagenized FMDV population P9 R it could be argued that all the components of its mutant spectrum were equally attenuated and that the virulent clones isolated were generated by mutations that arose during plaque formation. This unlikely possibility would imply that virulence-restoring mutations are produced in cell culture and not in the animal, again arguing in favor of suppressive effects of the complete quasispecies.

It may appear as if the results reported here are contradictory to those obtained with poliovirus in which the attenuation for mice was attained by a high fidelity mutant that produced a narrow mutant spectrum [11], [12], the opposite than the mutant spectrum of FMDV whose increased complexity resulted also in attenuation. This difference suggests that in the case of poliovirus, attenuation was associated with a deficiency in variability and adaptability, whereas in the case of FMDV, attenuation resulted from an excess of mutant genomes known to mediate interfering interactions. In both cases the results point to the need to examine the mutant spectrum of viral populations to understand the biological behavior of viruses [3], [4].

In summary, our resuts show that increased mutagenesis of a highly virulent RNA virus can reduce its virulence *in vivo* and that interefering interactions play an important role in the attenuation of the viral population. Thus, these findings encourage new investigations to optimize mutagen-based antiviral strategies for the treatment of RNA virus associated diseases.

## Materials and Methods

### Ethics Statement

All the experiments involving animals were approved by the ethical review committee at the Centro de Investigación en Sanidad Animal (CISA-INIA), following guidelines set forth the European Union (Directive 86/609/EEC).

### Viruses and cells

Procedures for infection of BHK-21 cell monolayers with FMDV in liquid medium [Dulbecco's modification of Eagle's medium (DMEM)] and for plaque assays in semisolid agar medium were carried out as previously described [36], [54]. FMDV C-S8c1 is a plaque-purified derivative of natural isolate C1-Sta Pau-Spain 70, a representative of the European subtype C1 FMDV [36]. Serial infections of FMDV C-S8c1 in BHK-21 cells in the presence of 5 mM ribavirin were carried out as previously described [28]. Briefly, 2×10^6^ BHK-21 cells were pre-incubated for 6 to 8 hours with 5 mM ribavirin (in 1% foetal calf serum (FCS), DMEM) to allow its penetration into cells and incorporation to cellular metabolic pathways [44]. Then, supernatants were removed, cells inoculated with FMDV C-S8c1 for 1 h at 37°C to allow virus adsorption, the monolayers washed, and DMEM (1% FCS) containing 5 mM ribavirin added to the infected cell monolayers. The infected cultures were incubated at 37°C for 24 hours or until complete cytopathology was observed, and the resulting supernatants were collected. The cytotoxicity of 5 mM ribavirin on BHK-21 cells has been previously assessed, with a reduction on cell viability of around 40% observed after 48 hours of treatment [7]. It must be noted that biological clone FMDV C-S8c1 used in the present study displays increased resistance (delayed extinction) than molecular clone pMT28 upon serial passages in the presence of ribavirin [7]; pMT28 is an infectious transcript that reproduces the consensus sequence of C-S8c1, except for having a shorter poly C tract [37], [55]. The mechanism that underlies the different resistance to ribavirin of biological clone C-S8c1 and molecular clone pMT28 has not been investigated.

Biological cloning of the C-S8c1 population passaged 9 times in the presence of ribavirin (P9 R) (Figure 1) was carried out by isolating virus from randomly chosen, well-isolated, individual virus plaques, avoiding biases due to plaque size, as previously described [16], [36], [56], [57].

### Mice and infections

Seven to eight weeks-old C57BL/6 female mice were purchased from Harlan Interfauna Ibérica S. L. and maintained at Centro de Investigación en Sanidad Animal (CISA-INIA) for one to two weeks to allow their acclimatization. Mice were inoculated subcutaneously in the left rear footpad (FP) with 50 µl of FMDV or culture medium (negative controls). The specific number of PFU inoculated and the experimental conditions are indicated in the corresponding footnote or Results section. Animals were examined daily for clinical symptoms. All the experiments with live animals were performed under the guidelines of the European Community (86/609/EEC) and were approved by the site ethical review committee.

### RNA extraction, cDNA synthesis, PCR amplification, and nucleotide sequencing

RNA was extracted from the supernatant of cell culture infections or from biological clones by treatment with Trizol (Invitrogen) according to the instructions of the manufacturer. Reverse transcription (RT) of FMDV RNA was performed using avian myeloblastosis virus reverse transcriptase (Promega) or Transcriptor reverse transcriptase (Roche), as specified by the manufacturer. For the determination of the consensus sequences (viral populations and biological clones), PCR amplification was carried out using the Expand high-fidelity polymerase system (Roche), as specified by the manufacturer. For the molecular cloning of individual viral RNA molecules from FMDV C-S8c1 and viral population P9 R, PCR amplification was carried out using PfuUltra DNA polymerase (Stratagene), due to its high copying fidelity [58], [59], as previously documented [60]. Nucleotide sequencing was carried as described in [57], [61].

### Molecular cloning

The genetic complexity of FMDV C-S8c1 and P9 R populations was analyzed by molecular cloning of the genomic region spanning residues 6800 to 7773, which corresponds to most of the viral polymerase gene (3D) (3D amino acids 65 to 453). PCR amplification was performed as indicated above with primers spanning FMDV residues 6610–6631 (forward orientation) and 7992–7953 (reverse orientation). cDNA products were purified and digested with the restriction enzymes HindIII (position 6667) and XhoI (position 7831) (New England BioLabs), and ligated to plasmid pGEM-4Z (Promega), previously digested with the same restriction enzymes, and treated with shrimp alkaline phosphatase (New England BioLabs). Transformation of *E. coli* DH5α, colony screening by PCR amplification, and nucleotide sequencing of individual clones were carried out as previously described [16]. Twenty-seven and 30 independent clones from populations P9 R and C-S8c1, respectively, were sequenced (a total of 26,000 to 29,000 nucleotides per viral population). DNA from positive colonies was amplified with the Expand high-fidelity polymerase system (Roche) according to the manufacturer's protocol, and sequenced as described above.

### Viral RNA quantification

FMDV RNA quantification was performed by real-time RT-PCR using the LightCycler instrument (Roche) and the RNA master SYBR green I kit (Roche), as specified by the manufacturer. Primers spanning FMDV genomic residues 4924 to 4944 (forward) and 5026 to 5047 (reverse) were used in the amplification [numbering of the FMDV genome residues is according to [61]. Quantification was relative to a standard curve obtained with known amounts of *in vitro* transcribed FMDV RNA, and was carried out as previously described [16], [55].

### Characterization of mutant spectra

The complexity of mutant spectra was characterized by determination of the mutation frequency and the normalized Shannon entropy, calculated with the formula Sn =  −[∑_i_ (pi x lnpi)]/ln N, in which pi is the proportion of each sequence of the mutant spectrum and N is the total number of sequences compared [62], as previously described [16], [41], [56], [63].
